# Physical activity, sleep, and fatigue in community dwelling Stroke Survivors

**DOI:** 10.1038/s41598-018-26279-7

**Published:** 2018-05-21

**Authors:** Anthony I. Shepherd, Richard Pulsford, Leon Poltawski, Anne Forster, Rod S. Taylor, Anne Spencer, Laura Hollands, Martin James, Rhoda Allison, Meriel Norris, Raff Calitri, Sarah G. Dean

**Affiliations:** 1 University of Portsmouth, Sport and Exercise Science, Portsmouth, PO1 2ER UK; 2 University of Exeter, Sport and Health Sciences, Exeter, EX1 2LU UK; 3 University of Exeter Medical School & PenCLAHRC, Exeter, EX1 2LU UK; 4 Academic Unit of Elderly Care, University of Leeds, Leeds, LS2 9LJ UK; 5 Royal Devon & Exeter Hospital, Exeter, EX2 5DW UK; 6 Torbay and Southern Devon Health and Care Trust, Torquay, TQ2 7TD UK; 7 Department of Clinical Sciences, Brunel University London, London, Uxbridge UB8 3PH UK

## Abstract

Stroke can lead to physiological and psychological impairments and impact individuals’ physical activity (PA), fatigue and sleep patterns. We analysed wrist-worn accelerometry data and the Fatigue Assessment Scale from 41 stroke survivors following a physical rehabilitation programme, to examine relationships between PA levels, fatigue and sleep. Validated acceleration thresholds were used to quantify time spent in each PA intensity/sleep category. Stroke survivors performed less moderate to vigorous PA (MVPA) in 10 minute bouts than the National Stroke guidelines recommend. Regression analysis revealed associations at baseline between light PA and fatigue (*p* = 0.02) and MVPA and sleep efficiency (*p* = 0.04). Light PA was positively associated with fatigue at 6 months (*p* = 0.03), whilst sleep efficiency and fatigue were associated at 9 months (*p* = 0.02). No other effects were shown at baseline, 6 or 9 months. The magnitude of these associations were small and are unlikely to be clinically meaningful. Larger trials need to examine the efficacy and utility of accelerometry to assess PA and sleep in stroke survivors.

## Introduction

In the five years after a stroke, an individuals’ odds of having a residual impairment is one in three^1^ which equates to more than 300,000 people in the UK alone^2^. Residual effects of stroke are the leading cause of chronic physiological and psychological disability^3^ and these effects can have deleterious consequences on an individual’s level of physical activity (PA)^4^ and sleep patterns^5^.

Traditional reporting methods for quantifying PA such as diaries and self-report tools have poor completion rates^6^, limited utility to distinguish between dimensions of PA^7^, are prone to recall bias, and have insufficient sensitivity to detect small changes in PA around interventions, particularly incidental light intensity PA which is sporadic and very difficult to recall accurately. These issues are potentially exacerbated in clinical populations such as stroke survivors^8^, especially if they have aphasia or impaired hand-writing function. Waterproof and continuous wear wrist worn (non-affected arm) accelerometers provide a viable option to quantify PA in stroke survivors, as to date there is limited evidence of sufficient quality to provide an accurate overview of PA in stroke survivors^9,10^.

UK national stroke guidelines recommend that stroke survivors participate in 150 minutes per week of moderate to vigorous PA (MVPA) in bouts of at least 10 minutes^11^. However, relatively few (42%) stroke survivors appear to meet the National Stroke guidelines for minutes per week of PA^4,12^. In addition to physical impairments, this may at least in part be explained by an individual’s perception of fatigue which is also known to reduce health-related quality of life^13^. Fatigue in stroke survivors is debilitating and is a common symptom, although its aetiology has yet to be elucidated^14^. If fatigued individuals reduce PA levels symptoms of fatigue often worsen^15^. In contrast, if individuals with fatigue perform exercise their symptoms often improve^16^ which suggests PA levels are interlinked with fatigue. Evidence also suggests fatigue may be related to sleep disorders in stroke survivors^13^.

Reductions in cognitive function following a stroke^3^ have been well characterised^17,18^ and have been linked to reductions in functional ability^19^. This may, at least in part, be due to lower levels of sleep quality in stroke survivors^5,20–22^. Reduction in sleep quality or prolonged sleep deprivation have been associated with a decline in health related quality of life, psychological wellbeing and physical performance in both healthy individuals and those with chronic disease^23,24^. PA has been shown to improve quality of sleep^25^. Interventions aimed at increasing PA and therefore sleep quality, may provide a novel pathway to improve quality of life and enable stroke survivors to regain independence.

Accelerometry is widely used to objectively quantify PA and sedentary periods such as sleep in active individuals^26–28^. However, to date there is limited evidence of sufficient quality to provide an accurate overview of PA or sleep in stroke survivors^9,10^. The gold standard assessment of sleep quality is polysomnography, however, this technique is both resource intensive and intrusive for the participant. Other methods include sleep diaries and self-reported questionnaires but these can lack accuracy^29^. Accelerometers are also increasingly being used as a tool to quantify sleep quality and efficiency^30,31^ as they are relatively inexpensive and are not burdensome.

In this paper we present exploratory analyses of data collected by accelerometers during the ReTrain trial^32^ - a randomised controlled trial of a physical rehabilitation training programme.

We aimed to determine the utility and acceptability of wrist worn accelerometers in community dwelling stroke survivors and to quantify stroke survivors’ levels of PA, sleep quantity and sleep efficiency. We then tested two hypotheses: (1) by utilising objectively measured PA (accelerometers), stroke survivor’s will perform lower amounts of PA compared to the recommendations and (2) that there will be associations, longitudinally at 6 and 9 months post intervention between PA, fatigue and sleep.

## Methods

### Design

A cross-sectional analysis of baseline data and predictive longitudinal analysis of 6 and 9 months follow up data from the participants in the ReTrain trial. The trial protocol^32^ and main results^33^ of the 12 week intervention and 6 and 9 month follow up have been published elsewhere and are summarised here.

### Participants

ReTrain aimed to recruit 48 community dwelling stroke survivors. Ethical approval was granted by the Cornwall and Plymouth NRES Committee (15/SW/0074) and the study was registered as a clinical trial on the ClinicalTrials.gov website, ID No. NCT02429180 (date of registration: 16/04/2015). This study was performed in accordance with the relevant guidelines and regulations outlined by the NRES committee. All participants provided written informed consent prior to entering the trial.

Inclusion criteria were: confirmation of stroke via their general practitioner, a minimum of 1 month post discharge from an NHS rehabilitation programme, ability to walk independently indoors with an aid but with (self-reported) difficulties with stairs or uneven surfaces, willingness to be randomised, and capacity to consent. Those younger than 18 years of age or with contraindications to moderate to vigorous PA were excluded^34^.

### Data collection

During an initial baseline visit an accelerometer, programmed to record movement data for 7 consecutive days, was fitted to the participant’s non-affected arm. Participants were also asked to complete a questionnaire booklet including the Fatigue Assessment Scale, a validated 10 item questionnaire with a 5 point Likert scale (with scores ranging from 10–50 with 10 representing no fatigue and 50 always fatigued)^35,36^. The minimal clinical meaningful difference (MCMD) is ≥4^37^. A minimum of 7 days later a second assessment visit was arranged to collect the accelerometer and questionnaire booklet. These data collection methods were repeated 6 and 9 months later.

### Assessment of physical activity and sleep

PA and sleep were assessed using wrist-worn accelerometers (GENEActiv, Activinsights, Kimbolton, Cambridge, UK). These devices have previously been validated for use in healthy adult populations^38^ and are extensively used in clinical studies. The GENEActiv accelerometers measured triaxial movement acceleration in gravity (*g*) units (1 g = 9.81 m/s^2^) at a frequency of 100 Hz continuously over a period of 7 days. The Euclidean norm (magnitude) of signals from the three axes minus 1 g (with negative numbers rounded zero) was used to quantify acceleration due to movement in mg (1 mg = 0.00981 m/s^2^)^39^. Following the measurement period, data were downloaded using manufacturers software and processed in R (R Core Team, Vienna, Austria) using the open source GGIR software package (http://cran.r-project.org).

Previously validated acceleration threshold values (in healthy adults)^38^ were used to quantify the time (minutes/day) spent on average in each intensity category: total PA, and separately for light, moderate, vigorous intensities and the composite category moderate-vigorous PA (MVPA). To facilitate comparison with current national stroke guidelines for PA^11^, average MVPA accumulated in bouts of at least 10 minutes was also calculated.

Sleep was determined from accelerometer data using an open source sleep detection algorithm in GGIR software. Sleep metrics derived using this method have demonstrated good levels of agreement with both self-report measures of sleep and polysomnography (the gold standard). The method of accelerometer based sleep quantification used here is described in detail elsewhere^40^. Briefly, wrist-worn triaxial accelerometers allow approximation of the angle of orientation of the arm relative to the horizontal plane. Periods of sleep are defined as nocturnal periods characterised by minimal movement frequency and magnitude of changes to the angle of the arm which does not include day time sleep. Time in bed was defined as the onset of the first period of sustained inactivity (as measured by changes of less than 5 degrees in a rolling 5 minute window) to the end of the last period of inactivity. Sleep duration is the sum of all recorded periods of sleep. Sleep efficiency can then be calculated as the sleep duration as a proportion of time in bed.

Periods of accelerometer non-wearing were identified using the range and standard deviation (SD) of acceleration values at each axis, calculated for rolling 60 minute windows. Non-wearing was indicated if the SD was <13.0 mg or if the range of values was <50 mg for two of the three axes. A full explanation of this method can be found elsewhere^39^. To allow effective assessment of habitual PA and sleep, measurement days when the accelerometer had been worn for less than 16 hours were excluded from the final analyses^39,41^. Participants who recorded less than 4 days of 16 hours were also excluded. A significant number of participants had hemiparesis, therefore the research team offered all participants in the trial assistance with fitting the accelerometers to their non-affected wrist (at baseline, 6 m and 9 m follow up).

### Statistical analysis

Data are presented as means and standard deviation (SD). Change scores from baseline to 6 months post randomisation and baseline to 9 months post randomisation were calculated for total PA, light PA, moderate PA, MVPA, and vigorous PA categories, time in bed, sleep time, sleep efficiency and self-reported fatigue. A series of multivariate linear regression models were fitted to estimate (1) the contribution of PA and sleep efficiency to fatigue (at baseline and change in these variables over 6 months and 9 months), and (2) the contribution of PA and fatigue to sleep efficiency (at baseline and change in these variables over 6 months and 9 months). All models were adjusted for gender, age, time since stroke, simplified modified Rankin scale (a scale to measure an individual’s degree of disability). Models assessing change in fatigue or sleep efficiency were also adjusted for baseline fatigue and sleep efficiency. Statistical significant differences were accepted at *p* < 0.05. Statistical analyses were performed on SPSS software version 22.0 (Chicago, IL). All data generated or analysed in this study are included in this published article (and its supplementary information files).

## Results

**Figure 1:**
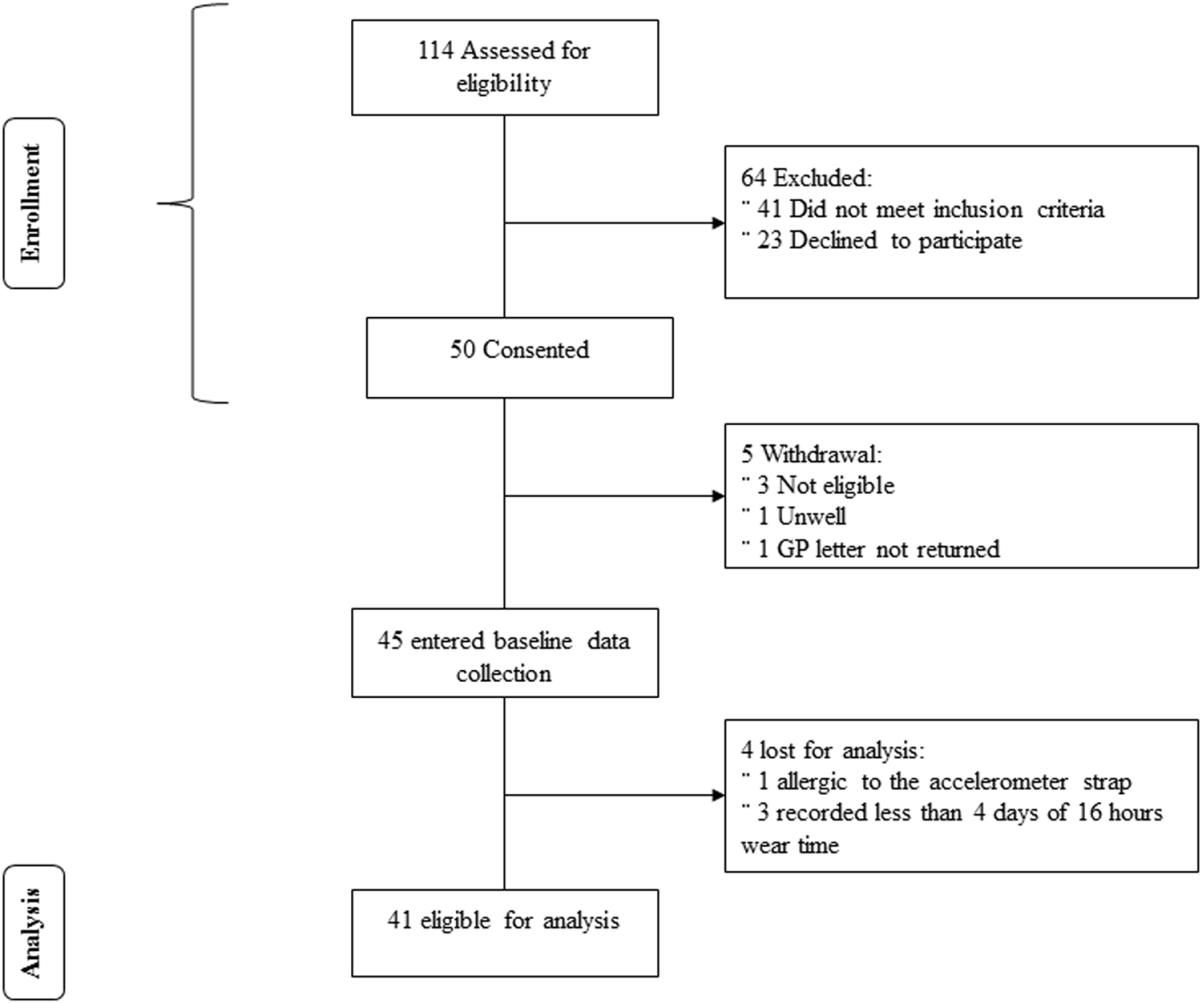
Flow diagram of the study.

Fifty stroke survivors consented to participate in the ReTrain trial (see Table 1 for participant characteristics). Forty-one participant data sets were eligible for analysis as four participants were excluded and one participant withdrew prior to randomisation. Three participants were excluded from the accelerometer analysis (for inadequate wear time) and one was lost to follow up (accelerometer not tolerated). For detailed analysis of participant flow through the study and reasons for withdrawal from the trial see Fig. 1.

**Table 1:** Characteristics of participants (n = 41) included in the final analysis.

Characteristics	Sub categories	Mean ± SD and/or percentage
Male n (%)		27 (66)
Age (years):		70 ± 11
Time since stroke (months)		60 ± 47
**Type of stroke n (%)**		
	Haemorrhagic	4 (10)
	Ischaemic	29 (71)
	Both	1 (2)
	Missing	7 (17)
**Co-morbidities, n (%)**		
	Hypertension	32 (78)
	Type 2 Diabetes Mellitus	7 (17)
	Depression	11 (27)
	Chronic Kidney Disease	2 (5)
	Asthma / COPD	6 (15)
	Other	7 (17)
MMSE		28 (68)
**Functional Disability (MRS) n (%)**		
	0	1 (2)
	1	3 (7)
	2	13 (32)
	3	24 (59)

MRS, Modified Rankin Scale. Some participants had multiple co-morbidities. Abbreviations, mini mental state examination (MMSE) and chronic obstructive pulmonary disease (COPD).

Compliance with the wrist worn accelerometers was very high. Participants averaged 4.0 ± 0.3 valid days of wear time for weekdays and 2.0 ± 0.2 days for weekend wear. Of the 45 participants that entered the baseline assessment period, 42 participants (93%) achieved or surpassed the wear time criteria of 16 hours / day, 4 days a week at each measurement stage.

### Description and quantification of PA in community dwelling stroke survivors

**Figure 2:**
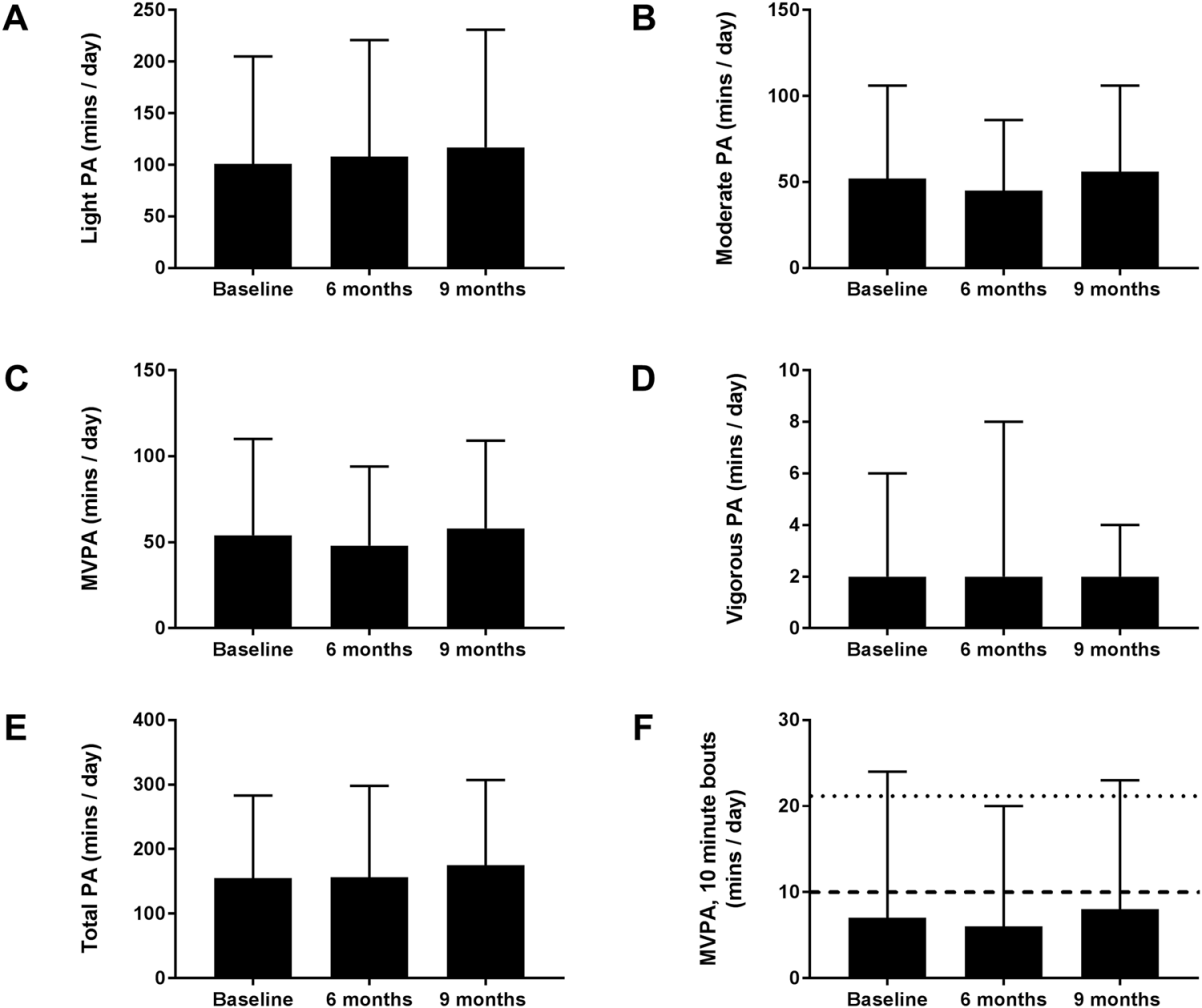
Average PA at baseline, 6 months and 9 months follow up. Mean ± SD in minutes for light PA (**A**), moderate PA (**B**), MVPA (**C**), Vigorous PA (**D**), Total PA (**E**) and MVPA in 10 minute bouts (**F**). For MVPA (F), the dashed line represents the minimum bout length and the dotted line represents the minimum level of MVPA in minutes per day for stroke survivors according to the National Stroke guidelines.

Our sample completed on average 155 minutes of total PA per day. Average minutes of total PA were split into light PA (101 ± 104 minutes), moderate PA (52 ± 54 minutes), vigorous PA (2 ± 4 minutes) and the combined variable MVPA (54 ± 56 minutes). On average (across measurement days) only 7 (±17) minutes per day of MVPA was accumulated in bouts of MVPA of over 10 minutes, see Fig. 2. Only 15% (n = 5) of our cohort achieved a 10 min bout of MVPA at baseline, which increased to 17% (n = 7) and 19% (n = 8) respectively at 6 and 9 months.

### Sleep quantity and efficiency

This cohort spent on average 10 hours in bed and 7.4 hours of that time asleep, equating to 74% sleep efficiency. Data from accelerometers are presented for sleep indices and self-reported fatigue in Table 2.

**Table 2:** Accelerometry based description of sleep in community dwelling stroke survivors. Fatigue, assessed via the fatigue assessment scale.

Measures	Mean	Quartiles			Change at 6 months (n = 37)	Change at 9 months (n = 32)
		25	50	75		
Sleep (hours)	7.4 ± 1.2	6.4	7.2	8.2	−0.0 ± 0.8	−0.2 ± 1.3
Time in bed (hours)	10 ± 1.2	9.2	10.1	11	−0.0 ± 0.8	−0.0 ± 1.0
Sleep Efficiency (%)	74 ± 10	66	75	80	0.0 ± 0.1	0.0 ± 0.1
Fatigue	26 ± 7	20	25	30	−2.7 ± 4.0	1.6 ± 5.1

Data are the mean ± SD or as quartiles.

### Exploratory analysis of associations between PA, sleep and fatigue

At baseline there was little evidence that PA was associated with fatigue (Table 3, model 1) or sleep efficiency (Table 4, model 1). Although there were statistically significant associations between light PA and fatigue (for every 10 minutes of light PA fatigue levels were 0.3 points lower) and MVPA and sleep efficiency (for every 10 minutes of MVPA sleep efficiency was 0.01 percentage points higher), these were very both small associations and unlikely to reflect any clinically meaningful change (MCMD ≤4 point change). There were also small levels of association between sleep efficiency and fatigue.

**Table 3:** Self-reported fatigue based on average total Light PA, average total MVPA and sleep efficiency: models examining (1) fatigue at baseline, (2) fatigue change at 6 months; (3) fatigue change at 9 months.

Measures	(1) Baseline^a^			(2) 6 Months^b^			(3) 9 Months^b^		
	Coefficient	95% CIs	*p*-value	Coefficient	95% CIs	*p*-value	Coefficient	95% CIs	*p*-value
Constant	55.37	29.88; 80.88	0.001	−1.89	−22.62; 18.84	0.85	−25.59	−55.45; 3.28	0.08
Trial Arm	−0.14	−4.67; 4.40	0.95	−2.16	−4.88; 0.56	0.11	1.74	−2.05; 5.54	0.35
Gender	−7.57	−12.75; −2.38	0.006	4.94	1.45; 8.42	0.01	4.03	−0.68; 8.73	0.09
Age	−0.08	−0.34; 0.18	0.56	−0.07	−0.23; 0.10	0.42	0.22	−0.04; 0.47	0.09
Time since stroke	−0.01	−0.06; 0.05	0.84	0.01	−0.03; 0.04	0.69	−0.03	−0.07; 0.02	0.24
sMRS	0.07	−4.63; 4.77	0.98	3.36	0.42; 6.57	0.04	0.71	−4.22; 5.63	0.77
Baseline Light PA	−0.03	−0.06; −0.01	0.02	0.01	−0.01; 0.03	0.29	0.01	−0.01; 0.03	0.32
Baseline MVPA	0.02	−0.03; 0.07	0.46	−0.03	−0.07; 0.01	0.17	0.01	−0.04; 0.06	0.66
Baseline Sleep efficiency	−25.14	−50.46; 0.19	0.05	0.39	−18.21; 18.99	0.97	8.18	−18.75; 35.10	0.53
Baseline Fatigue	—	—	—	0.05	−0.18; 0.28	0.65	0.16	−0.18; 0.50	0.34
Change in Sleep Efficiency	—	—	—	0.82	−20.18; 21.81	0.94	−44.01	−80319; −7.83	0.02
Change in Light PA	—	—	—	0.03	0.00; 0.05	0.03	0.01	−0.02; 0.04	0.55
Change in MVPA	—	—	—	0.02	−0.04; 0.08	0.45	−0.01	−0.11; 0.08	0.79
R^2^		0.35			0.47			0.54	

^a^Model adjusted for trial arm, gender, age, time since stroke, simplified modified rankin scale (sMRS), baseline sleep efficiency.

^b^Model adjusted for gender, age, time since stroke, simplified modified rankin scale (sMRS), baseline sleep efficiency, baseline fatigue.

**Table 4:** Sleep efficiency based on average total Light PA, average total MVPA and fatigue: models examining (1) sleep efficiency at baseline, (2) sleep efficiency change at 6 months; (3) sleep efficiency change at 9 months.

Measures	(1) Baseline^a^			(2) 6 Months^b^			(3) 9 Months^b^		
	Coefficient	95% CIs	*p*-value	Coefficient	95% CIs	*p*-value	Coefficient	95% CIs	*p*-value
Constant	0.77	0.44; 1.10	0.00	−0.01	−0.42; 0.42	0.98	−0.14	−0.49; 0.21	0.41
Trial Arm	−0.03	−0.09; 0.03	0.31	−0.00	−0.06; 0.06	0.98	0.01	−0.03; 0.06	0.54
Gender	−0.02	−0.10; 0.06	0.62	0.02	−0.06; 0.10	0.64	0.03	−0.02; 0.09	0.22
Age	0.00	0.00; 0.01	0.50	0.00	−0.00; 0.01	0.10	0.00	−0.00; 0.01	0.18
Time since stroke	0.00	0.00; 0.00	0.85	0.00	−0.00; 0.00	0.29	0.00	−0.00; 0.00	0.15
MRS	0.03	−0.03; 0.09	0.33	−0.06	−0.13; 0.01	0.07	−0.03	−0.08; 0.03	0.27
Baseline Light PA	0.00	0.00; 0.00	0.32	0.00	0.00; 0.00	0.17	0.00	0.00; 0.00	0.17
Baseline MVPA	0.001	0.00; 0.00	0.04	0.00	−0.00; 0.00	0.51	0.00	0.00; 0.00	0.42
Baseline Fatigue	−0.01	−0.01; 0.00	0.05	0.00	−0.00; 0.01	0.78	0.00	0.00; 0.01	0.19
Baseline Sleep efficiency	—	—	—	−0.24	−0.60; 0.12	0.18	−0.10	−0.38; 0.24	0.63
Change in Fatigue	—	—	—	0.00	−0.01; 0.01	0.94	−0.01	−0.01; −0.00	0.02
Change in Light PA	—	—	—	0.00	−0.00; 0.00	0.46	0.00	0.00; 0.00	0.76
Change in MVPA	—	—	—	−0.00	−0.00; 0.00	0.07	0.00	0.00; 0.00	0.88
R^2^		0.29			0.43			0.56	

^a^Model adjusted for trial arm, gender, age, time since stroke, simplified modified rankin scale (MRS), baseline sleep efficiency.

^b^Model adjusted for gender, age, time since stroke, simplified modified rankin scale (MRS), baseline sleep efficiency, baseline fatigue.

There was also little evidence that changes in PA levels were linked to changes in fatigue (Table 3, models 2 and 3) or sleep efficiency (Table 4, model 2 s and 3) at 6 or 9 months. However, we did find a statistically significant change in fatigue at 6 months suggesting that light PA was positively associated with fatigue: an increase of 10 minutes of light PA was associated with an increase in fatigue of 0.3 points (range of measure; 10–50). Sleep efficiency and fatigue were associated at 9 months but not at 6 months. At 9 months for every 1 percentage point increase in sleep efficiency there was a 44 point decrease (range of measure; 10–50) in fatigue (Table 3). A 1 point increase in fatigue was associated with a 0.01 percentage point decrease in sleep efficiency (Table 4). In addition, the effect of trial arm (control or intervention) was not significant, as expected, as the study was a pilot and was not set up to show statistically significant changes.

## Discussion

We have provided a robust quantification of PA and sleep in community dwelling stroke survivors. The cohort exhibited excellent compliance in wearing the devices and has provided the most robust and detailed objective analysis of stroke survivors PA and sleep efficiency to date. We found that stroke survivors performed significantly less MVPA in bouts of 10 minutes than recommended by the National Stroke guidelines at baseline and during follow up. We found small but statistically significant associations between PA, fatigue, and sleep efficiency.

While the cohort accumulated on average over 45 minutes per day of MVPA at each measurement point, the vast majority of this activity consisted of very short sporadic bouts of movement consistent with normal lifestyle activities^10,42^ rather than the more sustained (>10 minutes) bouts of volitional activity recommended for improvements in health for this patient group^11^. Less than one fifth (15–19% respectively) of our cohort perfumed a 10 minutes bout of MVPA at each data collection time point. In addition the cohort accumulated on average only 2 minutes of vigorous intensity PA per day across the week. Overall our data suggests that this cohort of stroke survivors accumulate the majority of their physical activity at the lower intensities, which may be insufficient to influence physical recovery or reduce risk of a subsequent stroke. Despite increasing evidence for the feasibility and acceptability of the use of wrist worn accelerometers in clinical studies^33^, evidence for accelerometer use to quantify PA in stroke survivors is sparse. Larger studies that utilise robust methodologies similar to this study (adequate wear time with patient friendly wrist-worn devices) are required to characterise PA in stroke survivors.

Changes in PA are linked with changes in fatigue profiles in individuals with chronic fatigue^15,16^, and in stroke survivors^43^. We showed that higher levels of light intensity PA were associated with reduced levels of fatigue at baseline and 9 months, but not at 6 months. We showed that for every 10 minutes of light PA fatigue levels were 0.3 points lower. Although these changes were statistically significant, we do not have sufficient statistical power in this study to confidently determine if they represent clinically meaningful changes. However, at 6 months, time spent in MVPA is reduced and light PA is increased. One suggestion is that the increase in light PA maybe displacing sleep or sedentary time. Regardless of the aforementioned limitations, the potential for light PA to be associated with reduced fatigue would suggest that further research into PA in stroke survivors is warranted. A larger definitive trial of ReTrain may be able to elucidate the effects of a training programme designed to improve functional ability, including investigating the impact on fatigue profiles in stroke survivors.

Stroke survivors have reduced sleep quality^5,20–22^ which can have profound effects on quality of life^23,24^. In line with evidence in healthy individuals^25^, we show the more time spent in PA or MVPA the more efficient a stroke survivor’s sleep is likely to be. However, this change is very small (with 10 minutes of MVPA sleep efficiency is higher by only 0.01 percentage points) and may not affect an individual’s quality of life. There was no association between light intensity PA and sleep efficiency which suggests that higher intensities of PA may be needed to obtain better quality (more efficient) sleep. Fatigue has previously been reported to be related to sleep disorders in stroke survivors^13^ and we show similar associations. However, the very wide confidence intervals around the measurement of fatigue (at both baseline and over time) should also be noted as they highlight an imprecise estimate of change, as would be expected from the small sample size.

Accelerometers were utilised to quantify both PA and sleep in this study. We demonstrated excellent compliance with the wrist worn accelerometers with 93% of participants surpassing the inclusion criteria for accelerometer wear time of 16 hours per day, 4 days a week. Earlier in the manuscript we highlight that other methods of quantifying sleep (polysomnography) can be resource intensive and intrusive to the participant. Due to a significant number of people having hemiparesis, the research team offered all participants in the trial assistance with fitting the accelerometers (in the participant’s home). All participants took up the offer and were visited on three occasions (at baseline, 6 m and 9 m follow up). This has significant resource use implications, however, this was mitigated as visits were scheduled alongside pre-planned assessment visits required by the pilot trial.

There are some limitations to our study. First, the ReTrain trial was not statistically powered to perform inferential analyses on study outcomes and the exploratory results from the regression models should be interpreted with caution. These analyses and processes have been utilised as a rehearsal for the definitive trial of ReTrain. Secondly, to date there are currently no established accelerometer cut-points to delineate activity intensity in stroke survivors. The cut-points used in the present study were developed using a non-clinical population which likely had a slightly higher level of cardiorespiratory fitness. This could increase the likelihood of misclassification of PA intensity. However in the present study this misclassification would have been consistent across time points and as such likely had little influence on estimates of change in PA from baseline. Future research is needed to establish PA intensity cut-points to better classify PA in stroke survivors. Larger powered studies aimed at establishing the clinically meaningful change in PA that is required to improve sleep efficiency are also required. A final limitation is that all participants had some degree of mobility impairment, whilst not all stroke survivors have an impairment this may at least in part explain the low levels of PA.

Using reliable, objective measures and with existing cut-points for non-clinical populations we showed that stroke survivors were performing less MVPA than recommended when compared against National Stroke guidelines, with less than one fifth performing these in more than 10 minute bouts. We found small but statistically significant levels of association between PA, fatigue and sleep efficiency. Light PA warrants further examination in stroke survivor’s as we show significant associations with fatigue and that the majority of stroke survivors do not meet the MVPA bout length recommendations. Larger studies are needed to examine the efficacy of utilising accelerometers to assess sleep in stroke survivors.

## Electronic supplementary material

Supplementary Dataset 1
