# Supplementary material for: Physical activity, sleep, and fatigue in community dwelling Stroke Survivors
Source: Sci Rep. 2018 May 21;8:7900. doi: 10.1038/s41598-018-26279-7 (PMC5962574; doi:10.1038/s41598-018-26279-7)
Supplement: Supplementary file 1 — Supplementary Dataset 1 [file 41598_2018_26279_MOESM1_ESM.docx]

***Title:*** Physical activity, sleep, and fatigue in community dwelling Stroke Survivors

*Authors:*

*Anthony I Shepherd^1^, Richard Pulsford^2^, Leon Poltawski^3^, Anne Forster^4^, Rod S Taylor^3^, Anne Spencer^3^, Laura Hollands^3^, Martin James^3,5^, Rhoda Allison^6^, Meriel Norris^7^, Raff Calitri^3^, Sarah G Dean^3^.

^1^ University of Portsmouth, Sport and Exercise Science, Portsmouth, PO1 2ER, UK. ^2^ University of Exeter, Sport and Health Sciences, Exeter, EX1 2LU, UK. ^3^ University of Exeter Medical School & PenCLAHRC, Exeter, EX1 2LU, UK. ^4^ Academic Unit of Elderly Care, University of Leeds, Leeds, LS2 9LJ, UK. ^5^ Royal Devon & Exeter Hospital, Exeter, EX2 5DW. ^6^ Torbay and Southern Devon Health and Care Trust, Torquay, TQ2 7TD, UK. ^7^ Department of Clinical Sciences, Brunel University, Middlesex, UB8 3PH, UK.

Corresponding author:

Dr Anthony Shepherd

Lecturer in Physical Activity, Exercise and Health

Department of Sport and Exercise Science

Faculty of Science, University of Portsmouth

Spinnaker Building

Cambridge Road

Portsmouth, PO1 2ER

Email: ant.shepherd@port.ac.uk

| **PID** | **CalculatedAge** | **Gender** | **TimeSinceStroke** | **SMRSScore** | **SMRS_cat** | **TrialArm** | **FAS_Total_0m** |
| --- | --- | --- | --- | --- | --- | --- | --- |
| 2 | 42 | 1 | 108 | 3 | 1 | 1.00 | 22 |
| 3 | 76 | 1 | 37 | 2 | 0 | 0.00 | 31 |
| 4 | 81 | 0 | 40 | 3 | 1 | 0.00 | 26 |
| 5 | 64 | 0 | 36 | 3 | 1 | 1.00 | 19 |
| 6 | 72 | 0 | 4 | 3 | 1 | 1.00 | 25 |
| 7 | 56 | 1 | 18 | 3 | 1 | 1.00 | 15 |
| 8 | 91 | 0 | 42 | 3 | 1 | 1.00 | 23 |
| 10 | 72 | 0 | 5 | 3 | 1 | 0.00 | 22 |
| 11 | 65 | 0 | 36 | 3 | 1 | 1.00 | 41 |
| 12 | 63 | 0 | 54 | 2 | 0 | 0.00 | 21 |
| 13 | 88 | 0 | 11 | 3 | 1 | 0.00 | 25 |
| 14 | 75 | 1 | 96 | 3 | 1 | 0.00 | 17 |
| 15 | 62 | 1 | 30 | 2 | 0 | 0.00 | 24 |
| 16 | 62 | 0 | 122 | 3 | 1 | 1.00 | 27 |
| 17 | 63 | 0 | 168 | 3 | 1 | 1.00 | 28 |
| 18 | 51 | 0 | 61 | 3 | 1 | 0.00 | 29 |
| 19 | 47 | 0 | 53 | 3 | 1 | 0.00 | 22 |
| 21 | 89 | 0 | 2 | 3 | 1 | 1.00 | 28 |
| 22 | 76 | 0 | 120 | 2 | 0 | 1.00 | 30 |
| 23 | 70 | 0 | 15 | 3 | 1 | 0.00 | 25 |
| 24 | 67 | 0 | 33 | 3 | 1 | 0.00 | 38 |
| 26 | 80 | 1 | 33 | 3 | 1 | 1.00 | 24 |
| 27 | 59 | 0 | 13 | 2 | 0 | 1.00 | 32 |
| 28 | 81 | 0 | 24 | 3 | 1 | 0.00 | 36 |
| 29 | 80 | 1 | 12 | 2 | 0 | 0.00 | 28 |
| 31 | 82 | 0 | 5 | 3 | 1 | 0.00 | 38 |
| 32 | 67 | 1 | 84 | 2 | 0 | 1.00 | 31 |
| 33 | 63 | 0 | 12 | 0 | 0 | 1.00 | 32 |
| 34 | 62 | 0 | 124 | 3 | 1 | 0.00 | 41 |
| 35 | 81 | 1 | 204 | 2 | 0 | 0.00 | 18 |
| 36 | 91 | 0 | 9 | 3 | 1 | 1.00 | 24 |
| 37 | 79 | 0 | 72 | 2 | 0 | 0.00 | 20 |
| 38 | 70 | 1 | 70 | 3 | 1 | 1.00 | 29 |
| 39 | 69 | 1 | 96 | 2 | 0 | 0.00 | 15 |
| 40 | 69 | 0 | 96 | 3 | 1 | 1.00 | 40 |
| 41 | 67 | 1 | 90 | 2 | 0 | 1.00 | 24 |
| 42 | 74 | 0 | 120 | 1 | 0 | 0.00 | 28 |
| 43 | 57 | 0 | 60 | 1 | 0 | 1.00 | 42 |
| 44 | 76 | 1 | 18 | 3 | 1 | 0.00 | 20 |
| 45 | 74 | 1 | 44 | 2 | 0 | 0.00 | 17 |
| 46 | 82 | 1 | 96 | 3 | 1 | 1.00 | 20 |
| 47 | 65 | 0 | 18 | 3 | 1 | 1.00 | 20 |
| 48 | 60 | 0 | 72 | 2 | 0 | 0.00 | 20 |
| 49 | 75 | 0 | 108 | 3 | 1 | 1.00 | 19 |
| 50 | 74 | 0 | 48 | 1 | 0 | 1.00 | 30 |

| **PID** | **FAS_Total_6m** | **FAS_Total_9m** | **validWEdays_0m** | **validWKdays_0m** | **validWEdays_6m** |
| --- | --- | --- | --- | --- | --- |
| 2 | 21 | 26 | 2 | 4 | 2 |
| 3 | 29 | 38 | 2 | 4 | 2 |
| 4 | 26 | 34 | 2 | 4 | 2 |
| 5 | 15 | 19 | 2 | 4 | 2 |
| 6 | 25 | 29 | 2 | 4 | 2 |
| 7 | 21 | 21 | 2 | 3 | 1 |
| 8 | 12 | 19 | 2 | 4 | 2 |
| 10 | 18 | 18 | 2 | 4 | 2 |
| 11 | 34 | . | 2 | 4 | 2 |
| 12 | 13 | 12 | 2 | 4 | 2 |
| 13 | 28 | 21 | 1 | 3 | 1 |
| 14 | . | 18 | 2 | 4 | . |
| 15 | 24 | 29 | 1 | 4 | 2 |
| 16 | 22 | 32 | 2 | 4 | 2 |
| 17 | 28 | 27 | 2 | 4 | 2 |
| 18 | 27 | 25 | 2 | 4 | 2 |
| 19 | 16 | 21 | 2 | 4 | 2 |
| 21 | 26 | 21 | 2 | 4 | 2 |
| 22 | 21 | 25 | 2 | 4 | 2 |
| 23 | 23 | 37 | 2 | 4 | 2 |
| 24 | 44 | 39 | 2 | 4 | 2 |
| 26 | 21 | 24 | 2 | 4 | 2 |
| 27 | 28 | 32 | 2 | 4 | 2 |
| 28 | 36 | 39 | 2 | 4 | 2 |
| 29 | 23 | 24 | 2 | 4 | 2 |
| 31 | . | . | 2 | 4 | . |
| 32 | 30 | 40 | 2 | 4 | 2 |
| 33 | 22 | 28 | 2 | 4 | 2 |
| 34 | 42 | 40 | 2 | 4 | 1 |
| 35 | 23 | 23 | 2 | 4 | 2 |
| 36 | . | . | 2 | 2 | . |
| 37 | 19 | 19 | 2 | 4 | 2 |
| 38 | 29 | 35 | 2 | 4 | 2 |
| 39 | 15 | 13 | 2 | 4 | 2 |
| 40 | . | . | 2 | 4 | . |
| 41 | 28 | 32 | 2 | 4 | 2 |
| 42 | 19 | 29 | 2 | 4 | 2 |
| 43 | 37 | 32 | 2 | 4 | 2 |
| 44 | . | . | 2 | 0 | . |
| 45 | 14 | 20 | 2 | 4 | 2 |
| 46 | 27 | 25 | 0 | 1 | . |
| 47 | 22 | 21 | 2 | 4 | 2 |
| 48 | 18 | 18 | 2 | 4 | 2 |
| 49 | 13 | 25 | 2 | 4 | 2 |
| 50 | 23 | 36 | 2 | 4 | 2 |

| **PID** | **validWKdays_6m** | **validWEdays_9m** | **validWKdays_9m** | **Av_Total_PA_0M** | **Av_Total_PA_6M** |
| --- | --- | --- | --- | --- | --- |
| 2 | 4 | 2 | 4 | 328.32 | 449.00 |
| 3 | 4 | 2 | 4 | 49.83 | 48.00 |
| 4 | 4 | 2 | 4 | 226.06 | 211.90 |
| 5 | 4 | 2 | 4 | 168.04 | 183.13 |
| 6 | 4 | 2 | 4 | 198.64 | 111.01 |
| 7 | 0 | 0 | 0 | 241.80 | . |
| 8 | 4 | . | . | 38.78 | 32.75 |
| 10 | 4 | 0 | 1 | 64.43 | 62.00 |
| 11 | 4 | 2 | 4 | 26.33 | 27.13 |
| 12 | 4 | 2 | 5 | 412.74 | 294.10 |
| 13 | 3 | 1 | 3 | . | . |
| 14 | . | 0 | 0 | 53.21 | . |
| 15 | 4 | 2 | 4 | 119.68 | 100.65 |
| 16 | 4 | 2 | 4 | 493.10 | 439.67 |
| 17 | 4 | 2 | 5 | 257.21 | 296.10 |
| 18 | 4 | 2 | 4 | 98.57 | 97.31 |
| 19 | 4 | 2 | 4 | 412.03 | 303.40 |
| 21 | 4 | . | . | 44.40 | 33.47 |
| 22 | 4 | 2 | 4 | 78.17 | 75.86 |
| 23 | 4 | 2 | 4 | 165.53 | 158.18 |
| 24 | 4 | 2 | 4 | 141.93 | 122.25 |
| 26 | 4 | 2 | 4 | 40.58 | 48.61 |
| 27 | 4 | 2 | 4 | 147.42 | 198.69 |
| 28 | 4 | 2 | 4 | 29.10 | 29.47 |
| 29 | 4 | 2 | 4 | 53.57 | 93.63 |
| 31 | . | . | . | 55.36 | . |
| 32 | 4 | 2 | 4 | 133.10 | 135.53 |
| 33 | 4 | 2 | 4 | 169.31 | 117.89 |
| 34 | 4 | 2 | 3 | 260.19 | 304.68 |
| 35 | 3 | 2 | 4 | 149.88 | 447.82 |
| 36 | . | . | . | . | . |
| 37 | 4 | 2 | 4 | 98.75 | 56.46 |
| 38 | 4 | 2 | 4 | 196.57 | 68.57 |
| 39 | 4 | 2 | 4 | 149.08 | 168.38 |
| 40 | . | . | . | 180.65 | . |
| 41 | 4 | 2 | 4 | 26.89 | 14.31 |
| 42 | 4 | 2 | 4 | 180.61 | 74.99 |
| 43 | 4 | 2 | 4 | 30.71 | 74.57 |
| 44 | . | . | . | . | . |
| 45 | 3 | 2 | 4 | 63.40 | 42.77 |
| 46 | . | . | . | . | . |
| 47 | 4 | 2 | 4 | 110.25 | 103.57 |
| 48 | 4 | 2 | 4 | 527.21 | 590.69 |
| 49 | 4 | 2 | 4 | 23.03 | 28.15 |
| 50 | 4 | 2 | 4 | 113.82 | 124.81 |

| **PID** | **Av_Total_PA_9M** | **Av_Light_PA_0M** | **Av_Light_PA_6M** | **Av_Light_PA_9M** |
| --- | --- | --- | --- | --- |
| 2 | 244.54 | 45.90 | 328.04 | 39.63 |
| 3 | 59.51 | 14.08 | 15.11 | 13.64 |
| 4 | 245.51 | 195.08 | 177.92 | 200.47 |
| 5 | 165.15 | 150.38 | 156.99 | 150.94 |
| 6 | 164.33 | 192.47 | 108.78 | 155.94 |
| 7 | . | 209.63 | . | . |
| 8 | . | 10.15 | 8.71 | . |
| 10 | . | 19.43 | 16.56 | . |
| 11 | 22.92 | 7.54 | 7.07 | 7.00 |
| 12 | 324.31 | 319.92 | 231.18 | 224.69 |
| 13 | . | . | . | . |
| 14 | . | 17.68 | . | . |
| 15 | 145.49 | 34.85 | 30.85 | 40.74 |
| 16 | 457.19 | 324.74 | 314.78 | 318.11 |
| 17 | 298.89 | 218.58 | 246.51 | 253.87 |
| 18 | 104.46 | 15.99 | 14.93 | 16.17 |
| 19 | 269.90 | 332.64 | 249.46 | 200.31 |
| 21 | . | 10.90 | 9.31 | . |
| 22 | 99.29 | 23.24 | 20.89 | 24.61 |
| 23 | 224.00 | 148.58 | 137.71 | 194.10 |
| 24 | 154.08 | 130.50 | 110.88 | 140.83 |
| 26 | 42.35 | 11.11 | 10.51 | 11.08 |
| 27 | . | 136.64 | 178.92 | . |
| 28 | 24.17 | 8.51 | 9.24 | 7.46 |
| 29 | 155.56 | 17.18 | 25.31 | 30.96 |
| 31 | . | 15.03 | . | . |
| 32 | 181.51 | 19.26 | 19.11 | 25.68 |
| 33 | 182.75 | 152.97 | 110.11 | 159.72 |
| 34 | 272.50 | 189.63 | 214.73 | 221.65 |
| 35 | 446.92 | 36.63 | 383.45 | 379.24 |
| 36 | . | . | . | . |
| 37 | 49.32 | 91.24 | 54.22 | 46.38 |
| 38 | 138.94 | 178.64 | 18.10 | 128.90 |
| 39 | 174.92 | 121.44 | 137.36 | 141.75 |
| 40 | . | 38.93 | . | . |
| 41 | 29.74 | 10.60 | 5.99 | 13.40 |
| 42 | 96.24 | 122.18 | 16.25 | 17.56 |
| 43 | 43.90 | 9.63 | 70.83 | 11.85 |
| 44 | . | . | . | . |
| 45 | 58.35 | 14.83 | 12.83 | 16.22 |
| 46 | . | . | . | . |
| 47 | 94.22 | 87.43 | 82.56 | 74.24 |
| 48 | 550.83 | 358.19 | 354.43 | 394.96 |
| 49 | 26.54 | 6.42 | 7.53 | 7.56 |
| 50 | 240.97 | 103.28 | 113.08 | 206.68 |

| **PID** | **Av_Moderate_PA_0M** | **Av_Moderate_PA_6M** | **Av_Moderate_PA_9M** | **Av_MVPA_PA_0M** |
| --- | --- | --- | --- | --- |
| 2 | 278.75 | 113.43 | 202.85 | 282.42 |
| 3 | 35.74 | 32.88 | 45.72 | 35.75 |
| 4 | 28.92 | 32.15 | 42.10 | 30.97 |
| 5 | 15.76 | 24.14 | 13.86 | 17.67 |
| 6 | 6.00 | 2.21 | 8.15 | 6.17 |
| 7 | 30.82 | . | . | 32.17 |
| 8 | 28.46 | 23.81 | . | 28.63 |
| 10 | 44.64 | 45.10 | . | 45.00 |
| 11 | 18.75 | 19.90 | 15.88 | 18.79 |
| 12 | 90.22 | 59.71 | 97.04 | 92.82 |
| 13 | . | . | . | . |
| 14 | 35.43 | . | . | 35.53 |
| 15 | 84.68 | 69.49 | 104.64 | 84.83 |
| 16 | 150.58 | 117.14 | 130.14 | 168.36 |
| 17 | 35.58 | 45.44 | 40.79 | 38.63 |
| 18 | 82.46 | 82.33 | 88.15 | 82.58 |
| 19 | 69.25 | 49.56 | 61.89 | 79.39 |
| 21 | 32.83 | 23.99 | . | 33.50 |
| 22 | 54.18 | 54.03 | 72.03 | 54.93 |
| 23 | 16.83 | 19.56 | 28.68 | 16.94 |
| 24 | 11.24 | 11.04 | 12.92 | 11.43 |
| 26 | 29.43 | 38.10 | 31.22 | 29.47 |
| 27 | 10.33 | 19.14 | . | 10.78 |
| 28 | 20.53 | 20.24 | 16.67 | 20.58 |
| 29 | 36.38 | 68.14 | 123.39 | 36.39 |
| 31 | 40.21 | . | . | 40.33 |
| 32 | 111.97 | 114.53 | 152.10 | 113.83 |
| 33 | 15.92 | 7.60 | 22.53 | 16.33 |
| 34 | 63.08 | 81.05 | 46.65 | 70.57 |
| 35 | 112.22 | 61.50 | 63.81 | 113.25 |
| 36 | . | . | . | . |
| 37 | 7.15 | 2.24 | 2.83 | 7.51 |
| 38 | 17.67 | 50.36 | 9.86 | 17.93 |
| 39 | 27.36 | 30.13 | 32.04 | 27.64 |
| 40 | 138.17 | . | . | 141.72 |
| 41 | 16.25 | 8.31 | 16.33 | 16.29 |
| 42 | 58.03 | 58.61 | 77.58 | 58.43 |
| 43 | 20.96 | 3.58 | 31.32 | 21.08 |
| 44 | . | . | . | . |
| 45 | 48.49 | 29.83 | 42.08 | 48.57 |
| 46 | . | . | . | . |
| 47 | 21.60 | 19.93 | 19.31 | 22.82 |
| 48 | 154.67 | 201.65 | 149.71 | 169.01 |
| 49 | 16.53 | 19.71 | 18.92 | 16.61 |
| 50 | 10.53 | 11.46 | 32.50 | 10.54 |

| **PID** | **Av_MVPA_PA_6M** | **Av_MVPA_PA_9M** | **Av_Vig_PA_0M** | **Av_Vig_PA_6M** | **Av_Vig_PA_9M** |
| --- | --- | --- | --- | --- | --- |
| 2 | 120.96 | 204.92 | 3.67 | 7.53 | 2.07 |
| 3 | 32.89 | 45.88 | 0.01 | 0.01 | 0.15 |
| 4 | 33.99 | 45.04 | 2.06 | 1.83 | 2.94 |
| 5 | 26.14 | 14.21 | 1.90 | 2.00 | 0.35 |
| 6 | 2.24 | 8.39 | 0.17 | 0.03 | 0.24 |
| 7 | . | . | 1.35 | . | . |
| 8 | 24.04 | . | 0.17 | 0.24 | . |
| 10 | 45.44 | . | 0.36 | 0.35 | . |
| 11 | 20.06 | 15.92 | 0.04 | 0.15 | 0.04 |
| 12 | 62.92 | 99.62 | 2.60 | 3.21 | 2.58 |
| 13 | . | . | . | . | . |
| 14 | . | . | 0.10 | . | . |
| 15 | 69.81 | 104.75 | 0.15 | 0.32 | 0.11 |
| 16 | 124.89 | 139.08 | 17.78 | 7.75 | 8.94 |
| 17 | 49.58 | 45.02 | 3.04 | 4.14 | 4.24 |
| 18 | 82.38 | 88.29 | 0.13 | 0.04 | 0.14 |
| 19 | 53.94 | 69.60 | 10.14 | 4.39 | 7.71 |
| 21 | 24.17 | . | 0.67 | 0.18 | . |
| 22 | 54.97 | 74.68 | 0.75 | 0.94 | 2.65 |
| 23 | 20.47 | 29.90 | 0.11 | 0.92 | 1.22 |
| 24 | 11.38 | 13.25 | 0.19 | 0.33 | 0.33 |
| 26 | 38.10 | 31.26 | 0.04 | 0.00 | 0.04 |
| 27 | 19.78 | . | 0.44 | 0.64 | . |
| 28 | 20.24 | 16.71 | 0.06 | 0.00 | 0.04 |
| 29 | 68.32 | 124.60 | 0.01 | 0.18 | 1.21 |
| 31 | . | . | 0.13 | . | . |
| 32 | 116.42 | 155.83 | 1.86 | 1.89 | 3.74 |
| 33 | 7.78 | 23.03 | 0.42 | 0.18 | 0.50 |
| 34 | 89.95 | 50.85 | 7.49 | 8.90 | 4.20 |
| 35 | 64.37 | 67.68 | 1.03 | 2.87 | 3.88 |
| 36 | . | . | . | . | . |
| 37 | 2.24 | 2.94 | 0.36 | 0.00 | 0.11 |
| 38 | 50.47 | 10.04 | 0.26 | 0.11 | 0.18 |
| 39 | 31.01 | 33.17 | 0.28 | 0.89 | 1.13 |
| 40 | . | . | 3.56 | . | . |
| 41 | 8.32 | 16.33 | 0.04 | 0.01 | 0.00 |
| 42 | 58.74 | 78.68 | 0.40 | 0.13 | 1.10 |
| 43 | 3.74 | 32.06 | 0.13 | 0.15 | 0.74 |
| 44 | . | . | . | . | . |
| 45 | 29.93 | 42.13 | 0.08 | 0.10 | 0.04 |
| 46 | . | . | . | . | . |
| 47 | 21.01 | 19.99 | 1.22 | 1.08 | 0.68 |
| 48 | 236.26 | 155.88 | 14.35 | 34.61 | 6.17 |
| 49 | 20.63 | 18.99 | 0.08 | 0.92 | 0.07 |
| 50 | 11.72 | 34.29 | 0.01 | 0.26 | 1.79 |

| **PID** | **Av_MVPA_PA_10MIN_0M** | **Av_MVPA_PA_10MIN_6M** | **Av_MVPA_PA_10MIN_9M** |
| --- | --- | --- | --- |
| 2 | 75.10 | 15.35 | 26.94 |
| 3 | 2.08 | 4.00 | 10.67 |
| 4 | 2.15 | 2.04 | 7.06 |
| 5 | 0.00 | 0.00 | 0.00 |
| 6 | 0.00 | 0.00 | 1.47 |
| 7 | 0.00 | . | . |
| 8 | 2.35 | 0.00 | . |
| 10 | 0.00 | 0.00 | . |
| 11 | 0.00 | 0.00 | 0.00 |
| 12 | 3.38 | 4.78 | 26.43 |
| 13 | . | . | . |
| 14 | 0.00 | . | . |
| 15 | 0.00 | 0.00 | 0.00 |
| 16 | 70.07 | 46.78 | 69.07 |
| 17 | 0.00 | 0.00 | 0.00 |
| 18 | 49.07 | 48.38 | 47.24 |
| 19 | 7.04 | 0.00 | 5.56 |
| 21 | 1.54 | 0.00 | . |
| 22 | 5.21 | 0.00 | 0.00 |
| 23 | 0.00 | 1.72 | 0.00 |
| 24 | 0.00 | 0.00 | 0.00 |
| 26 | 0.00 | 0.00 | 0.00 |
| 27 | 0.00 | 2.03 | . |
| 28 | 0.00 | 0.00 | 0.00 |
| 29 | 0.00 | 0.00 | 22.33 |
| 31 | 0.00 | . | . |
| 32 | 12.03 | 15.51 | 19.49 |
| 33 | 0.00 | 0.00 | 0.00 |
| 34 | 15.35 | 27.87 | 8.30 |
| 35 | 2.72 | 0.00 | 0.00 |
| 36 | . | . | . |
| 37 | 0.00 | 0.00 | 0.00 |
| 38 | 0.00 | 0.00 | 0.00 |
| 39 | 16.38 | 16.29 | 11.57 |
| 40 | 1.60 | . | . |
| 41 | 0.00 | 0.00 | 0.00 |
| 42 | 3.56 | 1.50 | 4.07 |
| 43 | 0.00 | 0.00 | 0.00 |
| 44 | . | . | . |
| 45 | 0.00 | 0.00 | 0.00 |
| 46 | . | . | . |
| 47 | 0.00 | 0.00 | 0.00 |
| 48 | 6.38 | 51.13 | 4.28 |
| 49 | 0.00 | 0.00 | 1.35 |
| 50 | 0.00 | 1.43 | 0.00 |

| **PID** | **Time_in_bed_0M** | **Time_in_bed_6M** | **Time_in_bed_9M** | **Sleep_0M** | **Sleep_6M** | **Sleep_9M** |
| --- | --- | --- | --- | --- | --- | --- |
| 2 | 8.92 | 9.27 | 9.88 | 7.51 | 8.16 | 8.28 |
| 3 | 10.84 | 10.51 | 10.72 | 7.03 | 7.69 | 8.22 |
| 4 | 9.28 | 9.76 | 9.22 | 8.19 | 8.57 | 8.53 |
| 5 | 10.93 | 10.83 | 11.33 | 7.22 | 7.29 | 7.35 |
| 6 | 10.69 | 10.70 | 9.85 | 7.18 | 7.66 | 7.24 |
| 7 | 11.21 | . | . | 6.86 | . | . |
| 8 | 9.71 | 10.57 | . | 7.22 | 7.94 | . |
| 10 | 10.62 | 10.90 | . | 9.44 | 9.63 | . |
| 11 | 11.94 | 12.11 | 10.69 | 9.54 | 6.88 | 3.05 |
| 12 | 9.56 | 8.37 | 8.17 | 6.21 | 6.80 | 5.41 |
| 13 | . | . | . | . | . | . |
| 14 | 9.83 | . | . | 6.02 | . | . |
| 15 | 9.83 | 10.57 | 9.58 | 7.85 | 8.19 | 7.37 |
| 16 | 7.88 | 8.22 | 8.85 | 6.32 | 6.53 | 6.16 |
| 17 | 11.15 | 10.31 | 11.57 | 7.59 | 7.29 | 8.03 |
| 18 | 10.10 | 10.37 | 10.23 | 8.39 | 7.96 | 7.96 |
| 19 | 9.21 | 9.78 | 10.42 | 7.21 | 7.60 | 8.02 |
| 21 | 11.26 | 11.50 | . | 8.76 | 9.59 | . |
| 22 | 10.64 | 11.19 | 9.18 | 7.03 | 8.46 | 6.60 |
| 23 | 11.52 | 11.75 | 11.26 | 9.86 | 9.36 | 8.33 |
| 24 | 7.61 | 9.65 | 11.05 | 4.36 | 5.09 | 5.72 |
| 26 | 12.52 | 12.29 | 11.81 | 10.04 | 10.49 | 9.24 |
| 27 | 8.78 | 9.59 | . | 6.49 | 7.67 | . |
| 28 | 10.23 | 10.18 | 10.20 | 8.21 | 7.64 | 8.11 |
| 29 | 8.74 | 8.82 | 8.75 | 6.39 | 7.00 | 7.46 |
| 31 | 10.49 | . | . | 6.23 | . | . |
| 32 | 10.14 | 10.75 | 10.05 | 7.51 | 8.22 | 7.72 |
| 33 | 10.83 | 8.88 | 8.76 | 5.74 | 5.12 | 5.48 |
| 34 | 9.90 | 9.87 | 10.45 | 6.29 | 6.20 | 6.93 |
| 35 | 7.65 | 7.36 | 7.52 | 6.87 | 6.28 | 6.60 |
| 36 | . | . | . | . | . | . |
| 37 | 11.47 | 10.29 | 11.20 | 8.21 | 7.61 | 8.15 |
| 38 | 9.76 | 10.10 | 10.58 | 7.49 | 7.52 | 8.09 |
| 39 | 8.57 | 9.35 | 8.26 | 7.36 | 7.50 | 7.32 |
| 40 | 8.61 | . | . | 6.58 | . | . |
| 41 | 10.00 | 9.26 | 10.26 | 6.93 | 6.52 | 6.42 |
| 42 | 9.67 | 8.27 | 9.20 | 7.81 | 7.41 | 7.28 |
| 43 | 10.40 | 9.71 | 10.01 | 6.14 | 6.16 | 6.32 |
| 44 | . | . | . | . | . | . |
| 45 | 10.57 | 10.22 | 11.16 | 8.15 | 8.14 | 7.98 |
| 46 | . | . | . | . | . | . |
| 47 | 11.48 | 10.77 | 11.10 | 10.07 | 9.13 | 9.76 |
| 48 | 8.02 | 8.86 | 7.08 | 6.15 | 6.43 | 5.92 |
| 49 | 11.08 | 10.43 | 11.35 | 7.32 | 4.55 | 6.52 |
| 50 | 11.24 | 10.84 | 11.02 | 8.03 | 6.93 | 7.83 |

| **PID** | **Sleep_efficency_0M** | **Sleep_efficency_6M** | **Sleep_efficency_9M** |
| --- | --- | --- | --- |
| 2 | 0.84 | 0.88 | 0.84 |
| 3 | 0.65 | 0.73 | 0.77 |
| 4 | 0.88 | 0.88 | 0.93 |
| 5 | 0.66 | 0.67 | 0.65 |
| 6 | 0.68 | 0.72 | 0.73 |
| 7 | 0.63 | . | . |
| 8 | 0.75 | 0.75 | . |
| 10 | 0.89 | 0.88 | . |
| 11 | 0.80 | 0.57 | 0.28 |
| 12 | 0.65 | 0.84 | 0.71 |
| 13 | . | . | . |
| 14 | 0.64 | . | . |
| 15 | 0.80 | 0.78 | 0.77 |
| 16 | 0.80 | 0.80 | 0.71 |
| 17 | 0.68 | 0.71 | 0.69 |
| 18 | 0.83 | 0.77 | 0.78 |
| 19 | 0.79 | 0.78 | 0.78 |
| 21 | 0.78 | 0.84 | . |
| 22 | 0.66 | 0.76 | 0.73 |
| 23 | 0.86 | 0.80 | 0.74 |
| 24 | 0.56 | 0.53 | 0.52 |
| 26 | 0.80 | 0.85 | 0.78 |
| 27 | 0.74 | 0.80 | . |
| 28 | 0.80 | 0.75 | 0.80 |
| 29 | 0.74 | 0.80 | 0.85 |
| 31 | 0.60 | . | . |
| 32 | 0.76 | 0.77 | 0.78 |
| 33 | 0.53 | 0.64 | 0.67 |
| 34 | 0.64 | 0.64 | 0.67 |
| 35 | 0.90 | 0.87 | 0.88 |
| 36 | . | . | . |
| 37 | 0.71 | 0.74 | 0.73 |
| 38 | 0.77 | 0.75 | 0.76 |
| 39 | 0.86 | 0.81 | 0.89 |
| 40 | 0.76 | . | . |
| 41 | 0.69 | 0.71 | 0.63 |
| 42 | 0.81 | 0.90 | 0.80 |
| 43 | 0.59 | 0.64 | 0.66 |
| 44 | . | . | . |
| 45 | 0.77 | 0.80 | 0.72 |
| 46 | . | . | . |
| 47 | 0.88 | 0.85 | 0.88 |
| 48 | 0.78 | 0.74 | 0.84 |
| 49 | 0.67 | 0.44 | 0.57 |
| 50 | 0.72 | 0.65 | 0.71 |

| **PID** | **SleepEfficiencyChange_0to6** | **SleepEfficiencyChange_0to9** | **FatigueChange_0to6** |
| --- | --- | --- | --- |
| 2 | 0.04 | 0.00 | -1.00 |
| 3 | 0.08 | 0.12 | -2.00 |
| 4 | 0.00 | 0.04 | 0.00 |
| 5 | 0.01 | -0.01 | -4.00 |
| 6 | 0.04 | 0.05 | 0.00 |
| 7 | . | . | 6.00 |
| 8 | 0.00 | . | -11.00 |
| 10 | 0.00 | . | -4.22 |
| 11 | -0.23 | -0.52 | -7.00 |
| 12 | 0.18 | 0.06 | -8.00 |
| 13 | . | . | 3.00 |
| 14 | . | . | . |
| 15 | -0.02 | -0.03 | 0.00 |
| 16 | -0.01 | -0.10 | -5.00 |
| 17 | 0.04 | 0.01 | 0.00 |
| 18 | -0.06 | -0.05 | -1.57 |
| 19 | -0.01 | -0.01 | -6.00 |
| 21 | 0.06 | . | -2.00 |
| 22 | 0.09 | 0.07 | -9.00 |
| 23 | -0.06 | -0.12 | -2.00 |
| 24 | -0.03 | -0.04 | 6.00 |
| 26 | 0.05 | -0.02 | -3.00 |
| 27 | 0.06 | . | -4.00 |
| 28 | -0.05 | -0.01 | 0.00 |
| 29 | 0.06 | 0.11 | -5.00 |
| 31 | . | . | . |
| 32 | 0.02 | 0.03 | -1.00 |
| 33 | 0.11 | 0.14 | -10.00 |
| 34 | 0.01 | 0.04 | 1.00 |
| 35 | -0.03 | -0.02 | 5.00 |
| 36 | . | . | . |
| 37 | 0.03 | 0.02 | -1.11 |
| 38 | -0.02 | -0.01 | 0.00 |
| 39 | -0.05 | 0.03 | 0.00 |
| 40 | . | . | . |
| 41 | 0.01 | -0.06 | 3.78 |
| 42 | 0.09 | -0.01 | -9.00 |
| 43 | 0.05 | 0.06 | -5.00 |
| 44 | . | . | . |
| 45 | 0.03 | -0.05 | -3.00 |
| 46 | . | . | 7.00 |
| 47 | -0.03 | 0.00 | 2.00 |
| 48 | -0.05 | 0.05 | -2.00 |
| 49 | -0.23 | -0.09 | -6.00 |
| 50 | -0.07 | -0.01 | -6.67 |

| **PID** | **FatigueChange_0to9** | **LIGHTChange_0to6** | **LIGHTChange_0to9** | **MVPAChange_0to6** |
| --- | --- | --- | --- | --- |
| 2 | 4.00 | 282.14 | -6.28 | -161.46 |
| 3 | 7.00 | 1.03 | -0.44 | -2.86 |
| 4 | 8.00 | -17.17 | 5.39 | 3.01 |
| 5 | 0.00 | 6.61 | 0.57 | 8.47 |
| 6 | 4.00 | -83.69 | -36.53 | -3.93 |
| 7 | 6.00 | . | . | . |
| 8 | -4.00 | -1.44 | . | -4.58 |
| 10 | -4.22 | -2.88 | . | 0.44 |
| 11 | . | -0.47 | -0.54 | 1.26 |
| 12 | -9.00 | -88.74 | -95.23 | -29.90 |
| 13 | -4.00 | . | . | . |
| 14 | 1.00 | . | . | . |
| 15 | 5.00 | -4.00 | 5.89 | -15.03 |
| 16 | 5.00 | -9.96 | -6.63 | -43.47 |
| 17 | -1.00 | 27.93 | 35.29 | 10.96 |
| 18 | -3.57 | -1.06 | 0.18 | -0.21 |
| 19 | -1.00 | -83.18 | -132.33 | -25.44 |
| 21 | -7.00 | -1.60 | . | -9.33 |
| 22 | -5.00 | -2.35 | 1.38 | 0.04 |
| 23 | 12.00 | -10.88 | 45.51 | 3.53 |
| 24 | 1.00 | -19.63 | 10.33 | -0.06 |
| 26 | 0.00 | -0.60 | -0.03 | 8.63 |
| 27 | 0.00 | 42.28 | . | 9.00 |
| 28 | 3.00 | 0.72 | -1.06 | -0.35 |
| 29 | -4.00 | 8.13 | 13.78 | 31.93 |
| 31 | . | . | . | . |
| 32 | 9.00 | -0.15 | 6.42 | 2.58 |
| 33 | -4.00 | -42.86 | 6.75 | -8.56 |
| 34 | -1.00 | 25.11 | 32.03 | 19.38 |
| 35 | 5.00 | 346.83 | 342.61 | -48.88 |
| 36 | . | . | . | . |
| 37 | -1.00 | -37.01 | -44.86 | -5.28 |
| 38 | 6.00 | -160.54 | -49.74 | 32.54 |
| 39 | -2.00 | 15.92 | 20.31 | 3.38 |
| 40 | . | . | . | . |
| 41 | 8.00 | -4.61 | 2.81 | -7.97 |
| 42 | 1.00 | -105.93 | -104.63 | 0.31 |
| 43 | -10.00 | 61.21 | 2.22 | -17.35 |
| 44 | . | . | . | . |
| 45 | 3.00 | -2.00 | 1.39 | -18.64 |
| 46 | 5.00 | . | . | . |
| 47 | 1.00 | -4.88 | -13.19 | -1.81 |
| 48 | -2.00 | -3.76 | 36.76 | 67.25 |
| 49 | 6.00 | 1.11 | 1.14 | 4.01 |
| 50 | 6.00 | 9.81 | 103.40 | 1.18 |

| **PID** | **MVPAChange_0to9** |
| --- | --- |
| 2 | -77.50 |
| 3 | 10.13 |
| 4 | 14.07 |
| 5 | -3.46 |
| 6 | 2.22 |
| 7 | . |
| 8 | . |
| 10 | . |
| 11 | -2.88 |
| 12 | 6.80 |
| 13 | . |
| 14 | . |
| 15 | 19.92 |
| 16 | -29.28 |
| 17 | 6.40 |
| 18 | 5.71 |
| 19 | -9.79 |
| 21 | . |
| 22 | 19.75 |
| 23 | 12.96 |
| 24 | 1.82 |
| 26 | 1.79 |
| 27 | . |
| 28 | -3.88 |
| 29 | 88.21 |
| 31 | . |
| 32 | 42.00 |
| 33 | 6.69 |
| 34 | -19.72 |
| 35 | -45.57 |
| 36 | . |
| 37 | -4.57 |
| 38 | -7.89 |
| 39 | 5.53 |
| 40 | . |
| 41 | 0.04 |
| 42 | 20.25 |
| 43 | 10.97 |
| 44 | . |
| 45 | -6.44 |
| 46 | . |
| 47 | -2.83 |
| 48 | -13.14 |
| 49 | 2.38 |
| 50 | 23.75 |

| **Data Dictionary** |  |  |
| --- | --- | --- |
| **Variable Name** | **Variable Label** | **Variable Value** |
| PID | Participant ID | |
| CalculatedAge | Age | |
| Gender | Gender | 0=male; 1=female |
| TimeSinceStroke | Time since stroke (months) | |
| SMRSScore | Simplified Modified Rankin Scale | |
| SMRS_cat | Simplified Modified Ranking Scale Category | 0-2=0; 3+=1 |
| TrialArm | Trial Arm | 0=control= 1=intervention |
| FAS_Total_0m | Fatigue Assessment Scale_Total baseline | |
| FAS_Total_6m | Fatigue Assessment Scale_Total 6 month | |
| FAS_Total_9m | Fatigue Assessment Scale_Total 9 month | |
| validWEdays_0m | Number of valid weekend days Baseline | |
| validWKdays_0m | Number of valid week days Baseline | |
| validWEdays_6m | Number of valid weekend days 6 month | |
| validWKdays_6m | Number of valid week days 6 month | |
| validWEdays_9m | Number of valid weekend days 9 month | |
| validWKdays_9m | Number of valid week days 9 month | |
| Av_Total_PA_0M | Average Total PA Baseline (mins) | |
| Av_Total_PA_6M | Average Total PA 6 month (mins) | |
| Av_Total_PA_9M | Average Total PA 9 Month (mins) | |
| Av_Light_PA_0M | Average Light PA Baseline (mins) | |
| Av_Light_PA_6M | Average Light PA 6 month (mins) | |
| Av_Light_PA_9M | Average Light PA 9 month (mins) | |
| Av_Moderate_PA_0M | Average Moderate PA Baseline (mins) | |
| Av_Moderate_PA_6M | Average Moderate PA 6 month (mins) | |
| Av_Moderate_PA_9M | Average Moderate PA 9 month (mins) | |
| Av_MVPA_PA_0M | Average MVPA PA Baseline (mins) | |
| Av_MVPA_PA_6M | Average MVPA PA 6 month (mins) | |
| Av_MVPA_PA_9M | Average MVPA PA 9 month (mins) | |
| Av_Vig_PA_0M | Average Vigorous PA Baseline (mins) | |
| Av_Vig_PA_6M | Average Vigorous PA 6 month (mins) | |
| Av_Vig_PA_9M | Average Vigorous PA 9 month (mins) | |
| Av_MVPA_PA_10MIN_0M | Average MVPA PA 10 min bouts Baseline (mins) | |

| **Variable Name** | **Variable Label** |
| --- | --- |
| Av_MVPA_PA_10MIN_6M | Average MVPA PA 10 min bouts 6 month (mins) |
| Av_MVPA_PA_10MIN_9M | Average MVPA PA 10 min bouts 9 month (mins) |
| Time_in_bed_0M | Time in Bed Baseline (hours) |
| Time_in_bed_6M | Time in Bed 6 month (hours) |
| Time_in_bed_9M | Time in Bed 9 month (hours) |
| Sleep_0M | Sleep Baseline (hours) |
| Sleep_6M | Sleep 6 month (hours) |
| Sleep_9M | Sleep 9 month (hours) |
| Sleep_efficency_0M | Sleep Efficiency Baseline (%) |
| Sleep_efficency_6M | Sleep Efficiency 6 month (%) |
| Sleep_efficency_9M | Sleep Efficiency 9 month (%) |
| SleepEfficiencyChange_0to6 | Sleep Efficiency change_Baseline - 6 month (%) |
| SleepEfficiencyChange_0to9 | Sleep Efficiency change_Baseline - 9 month (%) |
| FatigueChange_0to6 | Fatigue change_Baseline - 6 month |
| FatigueChange_0to9 | Fatigue change_Baseline - 9 month |
| LIGHTChange_0to6 | Average Light PA change_baseline 6 month (mins) |
| LIGHTChange_0to9 | Average Light PA change_baseline 9 month (mins) |
| MVPAChange_0to6 | Average MVPA PA change_baseline 6 month (mins) |
| MVPAChange_0to9 | Average MVPA PA change_baseline 9 month (mins) |
